# Supplementary material for: Phox2b-expressing neurons contribute to breathing problems in Kcnq2 loss- and gain-of-function encephalopathy models
Source: Nat Commun. 2023 Dec 5;14:8059. doi: 10.1038/s41467-023-43834-7 (PMC10698053; doi:10.1038/s41467-023-43834-7)
Supplement: Supplementary file 1 — Supplementary Figures [file 41467_2023_43834_MOESM1_ESM.pdf]

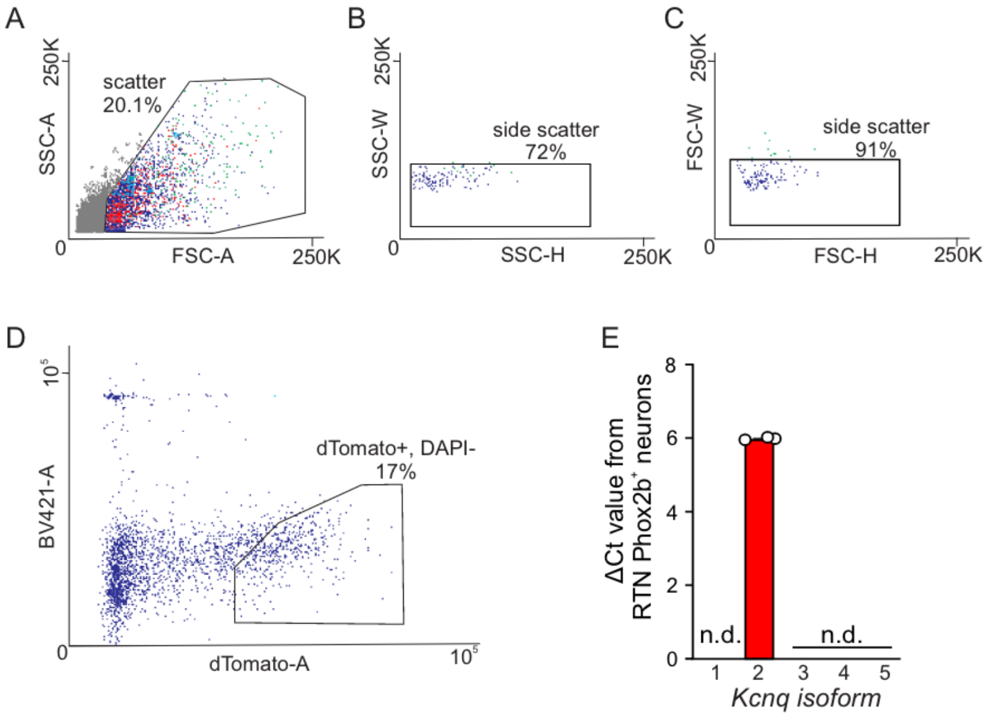

Supplemental Figure 1

Supplemental Figure 1. fluorescence-activated cell sorting (FACS) gating strategy to obtain an enriched population of tdT labeled Phox2b-expressing RTN neurons for qPCR analysis of *Kcnq(*1-5) transcripts. **A,** Scatter graph of gate used to filter out debris from the sample. **B**, Side scatter graph to gate for complexity/doublets. **C**, Forward scatter graph to gate for cell size. **D**, Scatter graph for DAPI and tdT. Cells were gated for positive tdT and low DAPI signal. **E**, FACS was used to obtain an enriched population of Phox2b-expressing cells and subsequent qPCR analysis of all five *Kcnq* isoforms shows that of the five members of the Kcnq family, we only detected Kcnq2 transcript in our sample of Phox2b-expressing ventral parafacial cells.

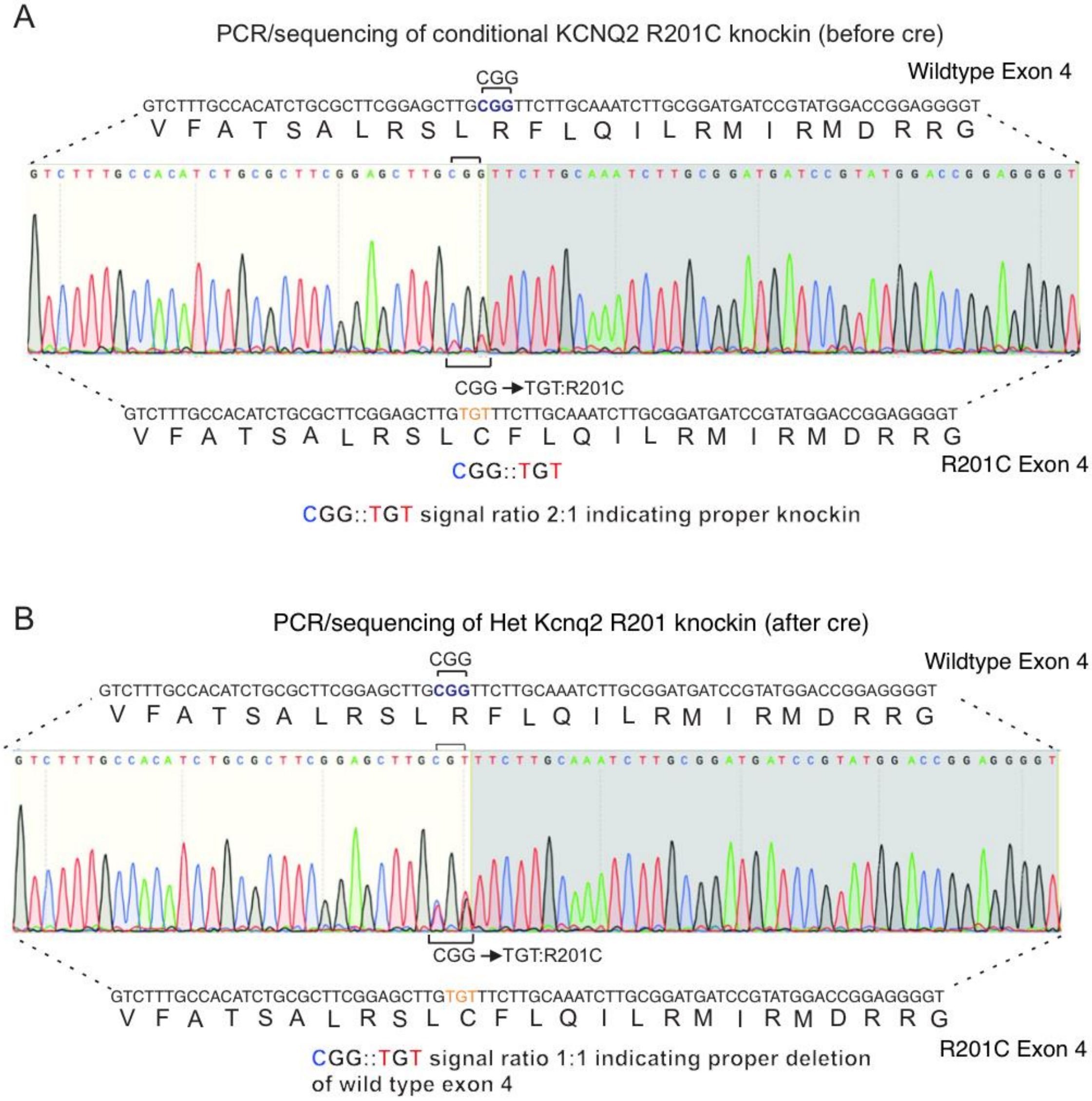

Supplemental Figure 2

Supplemental Figure 2. Validation of the Kcnq2 R201C knock-in mouse model. **A**, Sequencing of mouse genomic DNA of Kcnq2 R201C conditional heterozygous mouse show that in the absence of cre-recombinase, two wild-type copies of Kcnq2 exon 4 and one copy of the Kcnq2 R201C copy is present. **B**, genomic sequences of Kcnq2 R201C knock-in generated by crossing the conditional Kcnq2 ($Kcnq2^{R201C/+}$) to Hprt-cre mouse line shows the presence of one copy of wild-type Kcnq2 exon 4 and one copy of mutant Kcnq2 R201C exon 4. Note that the ratio of the thymine (T) and cytosine (C)/guanine(G) peaks at the 201 position is one-to-one consistent with the presence of one copy of wt and one copy of mutant allele. This is in contrast to the animals without cre-recombinase present.

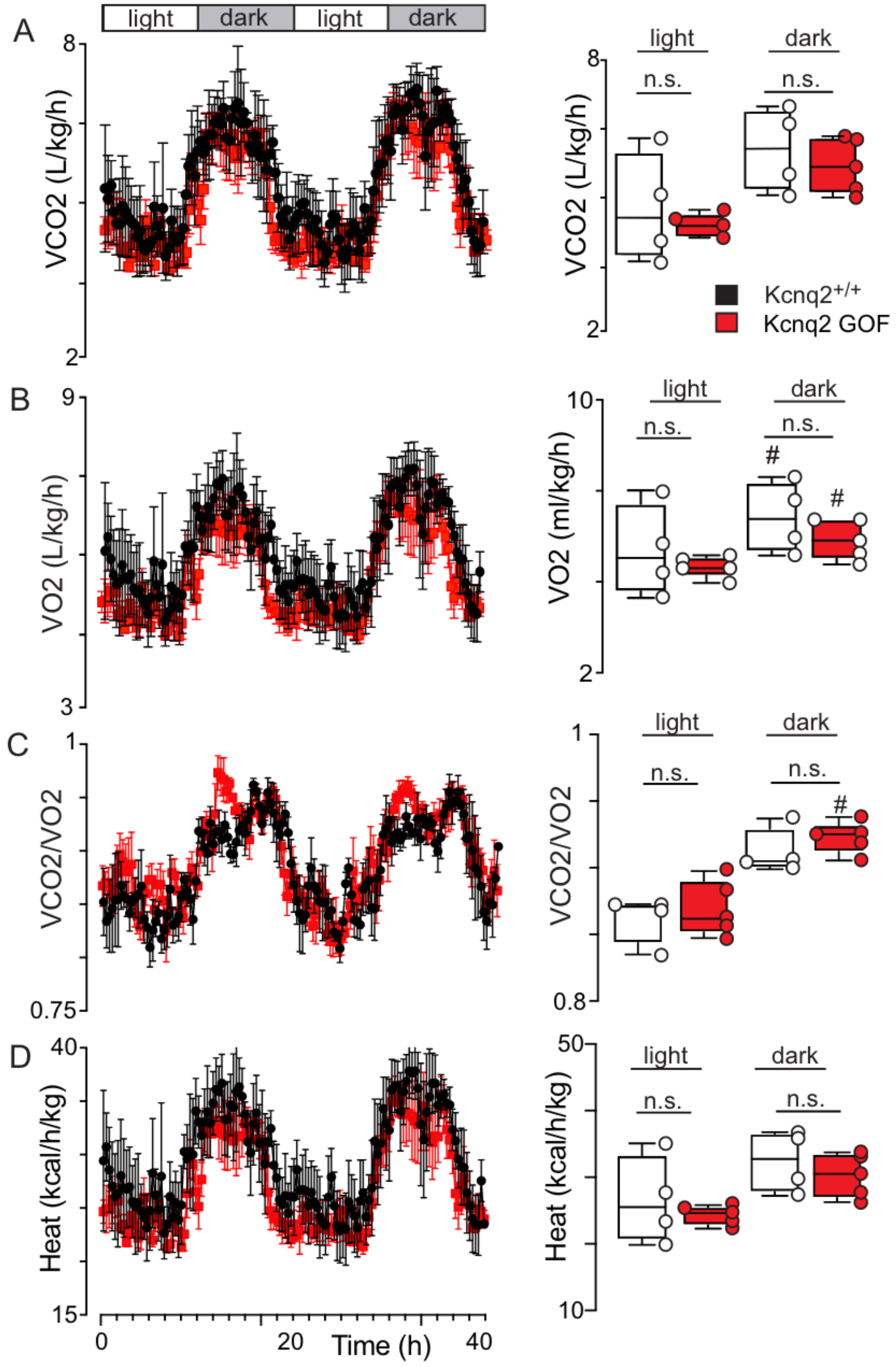

Supplemental Figure 3

Supplemental figure 3. Kcnq2$^{+/+}$ and Kcnq2 GOF mice show similar baseline metabolic activity. We characterized metabolic activity in Kcnq2 GOF (n=5) and Kcnq2$^{+/+}$ control (n=4) mice by measuring the volume of CO_2 produced relative to O_2 consumed and determining the (respiratory exchange ratio; VCO_2/VO_2) and calculating energy expenditure (heat; Kcal/hr/kg) during a 24-hour light/dark cycle. **A**, summary data plotted over time (left) and peak response during the light and dark cycles (right) show that Kcnq2 GOF and Kcnq2$^{+/+}$ have similar levels of baseline CO_2 production during the light ($T_7 = 0.68$, $p > 0.05$; Kcnq2$^{+/+}$ 4696 ± 599 and Kcnq2 GOF 4325 ± 116) and dark cycles ($T_7=0.68$, $p > 0.05$; Kcnq2$^{+/+}$ 5995 ± 457 and Kcnq2 GOF 5646 ± 272). **B**, summary data plotted over time (left) and peak response during the light and dark cycles (right) show that Kcnq2 GOF and Kcnq2$^{+/+}$ have similar levels of baseline O_2 consumption during the light ($T_7 = 1.65$, $p > 0.05$; Kcnq2$^{+/+}$ 0.34 ± 0.02 and Kcnq2 GOF 0.30 ± 0.01) and dark cycles ($T_7=2.1$, $p > 0.05$; Kcnq2$^{+/+}$ 0.42 ± 0.01 and Kcnq2 GOF 0.37 ± 0.01). **C**, summary data plotted over time (left) and peak response during the light and dark cycles (right) show that Kcnq2 GOF and Kcnq2$^{+/+}$ have similar respiratory exchange ratios during the light ($T_7 = 0.52$, $p > 0.05$; Kcnq2$^{+/+}$ 0.91 ± 0.01 and Kcnq2 GOF 0.92 ± 0.01) and dark cycles ($T_7=1.154$, $p>0.05$; Kcnq2$^{+/+}$ 0.91 ± 0.01 and Kcnq2 GOF 0.92 ± 0.01). **D**, summary data plotted over time (left) and peak response during the light and dark cycles show that energy expenditure (Heat; Kcal/h/kg) was similar between genotypes during the light ($T_7 = 0.74$, $p>0.05$; Kcnq2$^{+/+}$ 26.48 ± 3.29 and Kcnq2 GOF 24.25 ± 0.64) and dark cycles ($T_7 = 0.79$, $p>0.05$; Kcnq2$^{+/+}$ 32.32 ± 2.36 and Kcnq2 GOF 30.19 ± 1.5). Baseline metabolic activity was compared using an unpaired t-test.

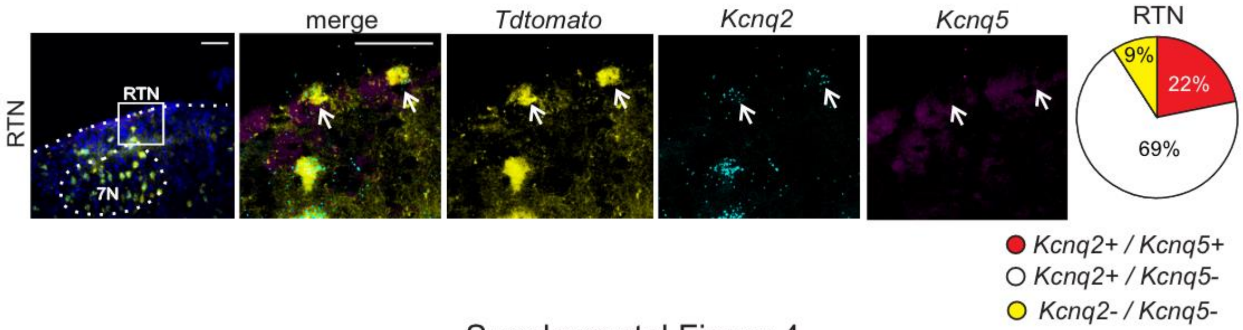

Supplemental Figure 4

Supplemental Figure 4. *Phox2b*-expressing parafacial neurons show modest expression of KCNQ5 transcript. **A**, Photomicrographs of coronal sections from the RTN region of *Phox2*$^{Cre/+}$::Ai14 reporter mice show tdT-labeled *Phox2b+* neurons (yellow) express *Kcnq2* transcript (green puncta) but no visible Kcnq signal. DAPI (blue) was used to label is label nuclei. Scale bar 100 µm. Insets (right), show fluorescent *in situ* hybridization labeling for the boxed region at a higher magnification where *Kcnq2* (green), *Kcnq5* (white), and *Phox2b*-tdT (yellow). Note that for display purposes we omitted DAPI from the inset images and used Phox2b to Phox2b to identify cell nuclei of interest. Scale bar 50 µm. **B**, summary of fluorescent *in situ* hybridization results (n= 3 mice, 30-40 days postnatal) show that 91% of tdT-*Phox2b* labeling in the parafacial region co-localizes with *Kcnq2* signal, and of these 22% also showed *KCNQ5* labeling, whereas 9% of tdT-*Phox2b* neurons in this region lacked both *Kcnq2* and *Kcnq5* signal.

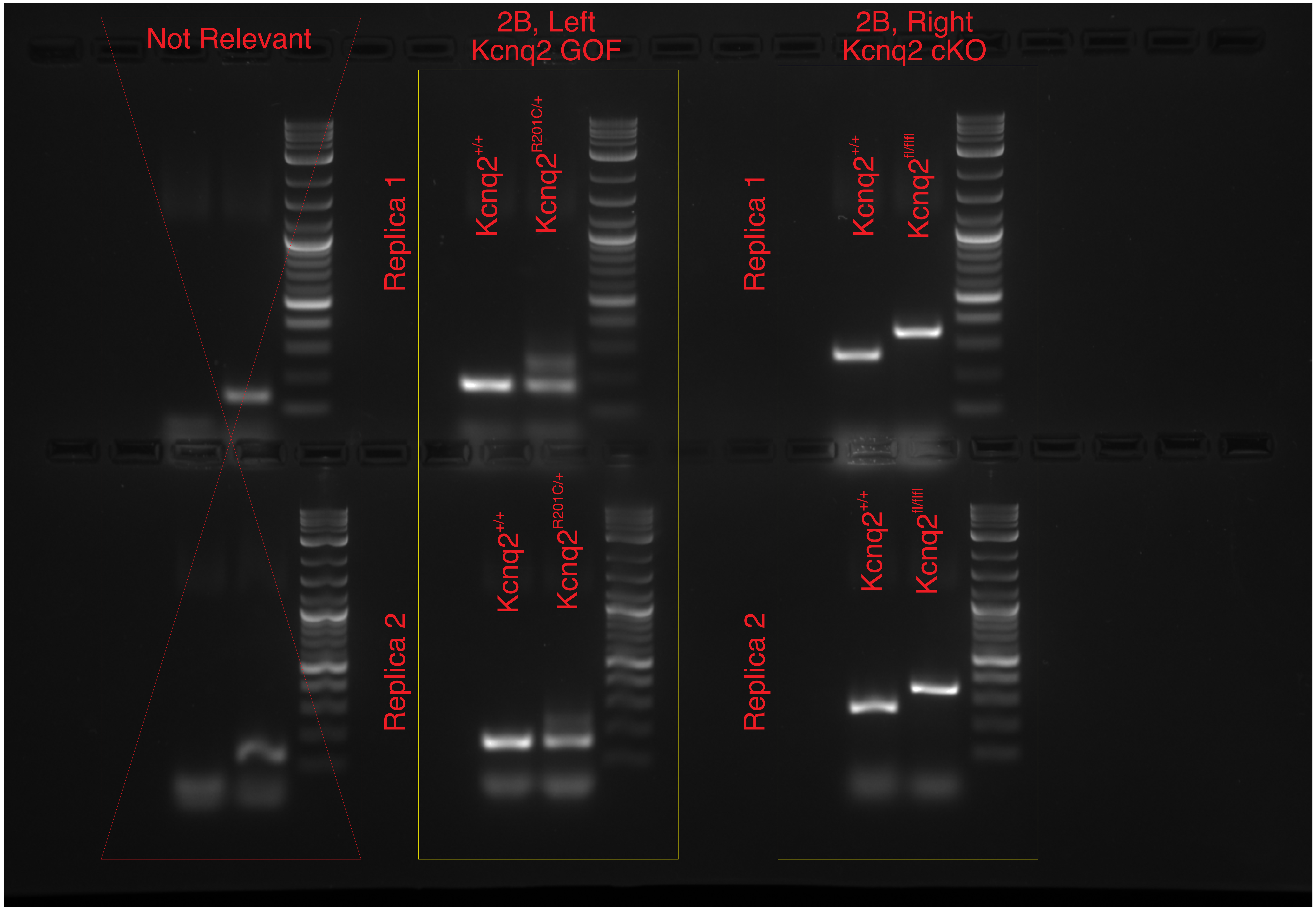

Supplemental Figure 5: Uncropped genotyping results of Kcnq2 GOF and Kcnq2 cKO mice. A. Uncropped PCR genotyping results of Kcnq2 GOF and Kcnq2 cKO mice from Figure 2B.
