# Supplementary material for: Phox2b-expressing neurons contribute to breathing problems in Kcnq2 loss- and gain-of-function encephalopathy models
Source: Nat Commun. 2023 Dec 5;14:8059. doi: 10.1038/s41467-023-43834-7 (PMC10698053; doi:10.1038/s41467-023-43834-7)
Supplement: Supplementary file 3 — reporting summary [file 41467_2023_43834_MOESM3_ESM.pdf]

nature portfolio

Corresponding author(s): Daniel Mulkey
Anastasios Tzingounis

Last updated by author(s): Nov 7, 2023

Reporting Summary

Nature Portfolio wishes to improve the reproducibility of the work that we publish. This form provides structure for consistency and transparency in reporting. For further information on Nature Portfolio policies, see our Editorial Policies and the Editorial Policy Checklist.

Please do not complete any field with "not applicable" or n/a. Refer to the help text for what text to use if an item is not relevant to your study.
For final submission: please carefully check your responses for accuracy; you will not be able to make changes later.

Statistics

For all statistical analyses, confirm that the following items are present in the figure legend, table legend, main text, or Methods section.

n/a	Confirmed	
☐	☒	The exact sample size (*n*) for each experimental group/condition, given as a discrete number and unit of measurement
☐	☒	A statement on whether measurements were taken from distinct samples or whether the same sample was measured repeatedly
☐	☒	The statistical test(s) used AND whether they are one- or two-sided<br>*Only common tests should be described solely by name; describe more complex techniques in the Methods section.*
☒	☐	A description of all covariates tested
☐	☒	A description of any assumptions or corrections, such as tests of normality and adjustment for multiple comparisons
☐	☒	A full description of the statistical parameters including central tendency (e.g. means) or other basic estimates (e.g. regression coefficient) AND variation (e.g. standard deviation) or associated estimates of uncertainty (e.g. confidence intervals)
☐	☒	For null hypothesis testing, the test statistic (e.g. *F*, *t*, *r*) with confidence intervals, effect sizes, degrees of freedom and *P* value noted<br>*Give P values as exact values whenever suitable.*
☒	☐	For Bayesian analysis, information on the choice of priors and Markov chain Monte Carlo settings
☒	☐	For hierarchical and complex designs, identification of the appropriate level for tests and full reporting of outcomes
☒	☐	Estimates of effect sizes (e.g. Cohen's *d*, Pearson's *r*), indicating how they were calculated

Our web collection on statistics for biologists contains articles on many of the points above.

Software and code

Policy information about availability of computer code

Data collection	Commercial software used for data collection are: Ponemah V5.32, ClampEx 11.03, QuantStudio Design & Analysis Software v1.5.1, Oxymax v5.54 CLAMS, FACSDiva 8.0, LAS X
Data analysis	Commercial software used for analysis are: ImageJ v2.0.0, QuantStudio Design & Analysis Software v1.5.1, FACSDiva 8.0, Clampfit 11.0.3, Graphpad PRISM 9, CED Spike2, Loupe Browser 5.

For manuscripts utilizing custom algorithms or software that are central to the research but not yet described in published literature, software must be made available to editors and reviewers. We strongly encourage code deposition in a community repository (e.g. GitHub). See the Nature Portfolio guidelines for submitting code & software for further information.

Data

Policy information about availability of data

All manuscripts must include a data availability statement. This statement should provide the following information, where applicable:

- Accession codes, unique identifiers, or web links for publicly available datasets
- A description of any restrictions on data availability
- For clinical datasets or third party data, please ensure that the statement adheres to our policy

The authors declare that all data supporting the claims in this study are available within the manuscript or its supporting information. Source data are included as a separate file. The raw scRNAseq data supporting findings in this study have been deposited in the GEO Repository with the primary accession code (GSE153172 ;

https://www.ncbi.nlm.nih.gov/geo/query/acc.cgi?acc=GSE153172). Additional raw data available upon request.

Research involving human participants, their data, or biological material

Policy information about studies with human participants or human data. See also policy information about sex, gender (identity/presentation), and sexual orientation and race, ethnicity and racism.

Reporting on sex and gender	NA
Reporting on race, ethnicity, or other socially relevant groupings	NA
Population characteristics	NA
Recruitment	NA
Ethics oversight	NA

Note that full information on the approval of the study protocol must also be provided in the manuscript.

Field-specific reporting

Please select the one below that is the best fit for your research. If you are not sure, read the appropriate sections before making your selection.

☒ Life sciences ☐ Behavioural & social sciences ☐ Ecological, evolutionary & environmental sciences

Life sciences study design

All studies must disclose on these points even when the disclosure is negative.

Sample size	In-vivo sample size calculation were done with an alpha of 0.05 for 80% power were appropriate. Sample sizes for cellular experiments were not predetermined; they were based on effect size and variability of the data for each experiment.
Data exclusions	Inclusion criteria for cellular experiments are included in the text, cell that did not meet this criteria were excluded from analysis. For in-vivo experiments, mice that exhibited excessive behavioral artifacts were excluded. A statistical analysis (Grubbs' test) was used to identify and exclude outliers in both cellular and behavioral experiments.
Replication	Specific number of animals and trials are detailed in text, methods, and/or legends.
Randomization	We randomized the use of control and experimental animals for every experiment.
Blinding	The experimenter was not blind to genotype during data acquisition. The reason for this is twofold. First, we obtain knockout and knock-in mice at a much lower frequency then control mice; therefore, we determined genotype prior to assessment of behavior to ensure that the appropriate number of animals of each genotype were included in each experiment. Second, for cellular experiments knock-in mice were readily identified by expression of a fluorescent reporter so it was not possible to perform these experiments blind. Data analysis was performed blind.

Reporting for specific materials, systems and methods

We require information from authors about some types of materials, experimental systems and methods used in many studies. Here, indicate whether each material, system or method listed is relevant to your study. If you are not sure if a list item applies to your research, read the appropriate section before selecting a response.

Materials & experimental systems

n/a	Involved in the study	
	☒	Antibodies
☒		Eukaryotic cell lines
☒		Palaeontology and archaeology
	☒	Animals and other organisms
☒		Clinical data
☒		Dual use research of concern
☒		Plants

Methods

n/a	Involved in the study	
☒		ChIP-seq
	☒	Flow cytometry
☒		MRI-based neuroimaging

Antibodies

Antibodies used	Primary Antibodies:<br>Goat anti-Phox2b, 1:100, R&D Systems, Cat# AF4940, Lot# CBDC0218041<br>Rabbit anti-Lucifer yellow, 1:500, ThermoFisher, Cat# A-5750, Lot# 2303158<br>Rabbit anti-Kcnq2, 1:250, Cat# PA1-929, Lot# YB370589<br>Rabbit anti-dsRed, 1:400, Takara Biosciences, Cat# AB_10013483, Lot# 1904182<br><br>Secondary Antibodies (all 1:500 dilution, Jackson ImmunoResearch):<br>Donkey anti-goat 647, Cat# 705-605-003, Lot# 155166<br>Donkey anti-rabbit 488, Cat# 711-545-152, Lot# 164289<br>Donkey anti-rabbit Cy3, Cat# 711-165-152, Lot# 166393
Validation	Goat anti-Phox2b antibody has been validated for use in ICC and ELISA as indicated on the manufacturer website https: www.rndsystems.com/products/human-mouse-phox2b-antibody_af4940. We have previously used this antibody for IHC applications (PMID: 31025941).<br><br>Rabbit-anti Lucifer yellow antibody has extensive literature in amplification of Lucifer yellow signal in a variety of preparations and validation for post-hoc IHC on filled cells can be found on the manufacturer website: https://www.thermofisher.com/antibody/product/Lucifer-Yellow-Antibody-Polyclonal/A-5750<br><br>Rabbit anti-Kcnq2 antibody validation is available on the manufacturer website (https://www.thermofisher.com/antibody/product/KCNQ2-Antibody-Polyclonal/PA1-929) and has been validated by IHC in this study.<br><br>Rabbit anti-dsRed antibody has been used extensively to boost signal from tdTomato and references for validation are available on the manufacturer website: https://www.takarabio.com/products/antibodies-and-elisa/fluorescent-protein-antibodies/red-fluorescent-protein-antibodies<br><br>Secondary antibodies from Jackson ImmunoResearch have been extensively used in the literature. More information is available at: https://www.jacksonimmuno.com/technical/products/groups/whole-igg

Animals and other research organisms

Policy information about studies involving animals; ARRIVE guidelines recommended for reporting animal research, and Sex and Gender in Research

Laboratory animals	All mice used in this study were maintained on a C57Bl/6 background. Animals were housed on a 12 hour light-dark cycles and had standard rodent chow and water ad libitum.<br><br>C57Bl/6 (JAX # 000664)<br>Kcnq2-R201C (this study)<br>Kcnq2-fl/fl (JAX # 036075)<br>Phox2b-Cre (JAX # 016223)<br>Ai14 (JAX # 0079144)<br>For imunohistochemistry, P20-50 mice were used<br>For cellular experiments, P7-P11 mire were used.<br>For behavioral experiments, adult animals P30-P50
Wild animals	no wild animals were used in this study
Reporting on sex	Males and females were used in this study; however, experiments were not designed or powered to detect sex differences. Within a genotype, all whole animal behaviors of interest were statistically similar between sexes so male and female mice were pooled for analysis.
Field-collected samples	no feild collected samples were used in this study.
Ethics oversight	University of Connecticut Institutional Animal Care and Use Committee (IACUC)

Note that full information on the approval of the study protocol must also be provided in the manuscript.

Flow Cytometry

Plots

Confirm that:

☒ The axis labels state the marker and fluorochrome used (e.g. CD4-FITC).

☒ The axis scales are clearly visible. Include numbers along axes only for bottom left plot of group (a 'group' is an analysis of identical markers).

☒ All plots are contour plots with outliers or pseudocolor plots.

☒ A numerical value for number of cells or percentage (with statistics) is provided.

Methodology

Sample preparation	Sample preparation for pooled qPCR are detailed in the methods section. In short, adult Phox2b-Cre::Tdt mice were used to make a single cell suspension from the RTN. 3 animals were used to satisfy biological replicates; 3 technical replicates per sample were used for each Taqman probe.
Instrument	BD FACSAria II Cell Sorter
Software	BD FACSDiva 8.0
Cell population abundance	Phox2b(TdT+) cells made up 17% of the total parent population of live (DAPI Negative) cells in a 1,956 event sample (data available upon request)
Gating strategy	A 10k threshold was applied to filter debris and dead cells from suspension. Single cells were then selected based on side and forward scatter ad then gated for the absence of DAPI. Finally, cells were gated on the presence of Tdtomato fluorescence in 4-way purity mode.

☒ Tick this box to confirm that a figure exemplifying the gating strategy is provided in the Supplementary Information.

This checklist template is licensed under a Creative Commons Attribution 4.0 International License, which permits use, sharing, adaptation, distribution and reproduction in any medium or format, as long as you give appropriate credit to the original author(s) and the source, provide a link to the Creative Commons license, and indicate if changes were made. The images or other third party material in this article are included in the article's Creative Commons license, unless indicated otherwise in a credit line to the material. If material is not included in the article's Creative Commons license and your intended use is not permitted by statutory regulation or exceeds the permitted use, you will need to obtain permission directly from the copyright holder. To view a copy of this license, visit http://creativecommons.org/licenses/by/4.0/
