# Supplementary material for: Phox2b-expressing neurons contribute to breathing problems in Kcnq2 loss- and gain-of-function encephalopathy models
Source: Nat Commun. 2023 Dec 5;14:8059. doi: 10.1038/s41467-023-43834-7 (PMC10698053; doi:10.1038/s41467-023-43834-7)
Supplement: Supplementary file 4 — source data [file 41467_2023_43834_MOESM4_ESM.pdf]

Supplemet Fig 3 A			
VCO2			
Light		Dark	
Control	Kcnq2 GOF	Control	Kcnq2 GOF
3541.07576	4672.57576	4999.28788	6293.62121
4002.22727	4338.80303	5467.95455	5148.66667
4994.07576	4061.84848	6569.69697	4948.24242
6248.25758	4471.81818	6946.68182	6222.40909
	4081.16667		5621.01515

Supplemet Fig 3 C			
RER			
Light		Dark	
Control	Kcnq2 GOF	Control	Kcnq2 GOF
0.86815152	0.85481818	0.90565152	0.92560606
0.8339697	0.89645455	0.90428788	0.9380303
0.87087879	0.86068182	0.89890909	0.92510606
0.87154545	0.84631818	0.93693939	0.90540909
	0.8815		0.91887879

Supplemet Fig 3 B			
VO2			
Light		Dark	
Control	Kcnq2 GOF	Control	Kcnq2 GOF
4177.06061	5426.90909	5440.0101	6495.47475
4943.54545	5048.35354	5984.20202	5511.32323
5750.83838	4619.09091	7043.65657	5183.80808
7334.0202	5300.79798	7752.12121	6495.0202
	5052.69697		5880.63636

Supplemet Fig 3 D			
Heat			
Light		Dark	
Control	Kcnq2 GOF	Control	Kcnq2 GOF
19.8469697	24.6136364	27.1075758	27.4681818
23.1787879	22.269697	29.5742424	26.1424242
27.8530303	25.2287879	35.8409091	33.1984848
35.0742424	23.35	36.7924242	30.4378788
	25.8242424		33.7075758
